# Sugariness prediction of *Syzygium samarangense* using convolutional learning of hyperspectral images

**DOI:** 10.1038/s41598-022-06679-6

**Published:** 2022-02-17

**Authors:** Chih-Jung Chen, Yung-Jhe Yan, Chi-Cho Huang, Jen-Tzung Chien, Chang-Ting Chu, Je-Wei Jang, Tzung-Cheng Chen, Shiou-Gwo Lin, Ruei-Siang Shih, Mang Ou-Yang

**Affiliations:** 1 National Yang Ming Chiao Tung University, Hsinchu, Taiwan; 2 Fengshan Tropical Horticultural Experiment Branch, Kaohsiung, Taiwan; 3 Feng Chia University, Taichung, Taiwan; 4 National Taiwan Ocean University, Keelung, Taiwan

**Keywords:** Computational biology and bioinformatics, Plant sciences, Engineering, Mathematics and computing, Optics and photonics

## Abstract

Sugariness is one of the most important indicators to measure the quality of *Syzygium samarangense*, which is also known as the wax apple. In general, farmers used to measure sugariness by testing the extracted juice of the wax apple products. Such a destructive way to measure sugariness is not only labor-consuming but also wasting products. Therefore, non-destructive and quick techniques for measuring sugariness would be significant for wax apple supply chains. Traditionally, the non-destructive method to predict the sugariness or the other indicators of the fruits was based on the reflectance spectra or Hyperspectral Images (HSIs) using linear regression such as Multi-Linear Regression (MLR), Principal Component Regression (PCR), and Partial Least Square Regression (PLSR), etc. However, these regression methods are usually too simple to precisely estimate the complicated mapping between the reflectance spectra or HSIs and the sugariness. This study presents the deep learning methods for sugariness prediction using the reflectance spectra or HSIs from the bottom of the wax apple. A non-destructive imaging system fabricated with two spectrum sensors and light sources is implemented to acquire the visible and infrared lights with a range of wavelengths. In particular, a specialized Convolutional Neural Network (CNN) with hyperspectral imaging is proposed by investigating the effect of different wavelength bands for sugariness prediction. Rather than extracting spatial features, the proposed CNN model was designed to extract spectral features of HSIs. In the experiments, the ground-truth value of sugariness is obtained from a commercial refractometer. The experimental results show that using the whole band range between 400 and 1700 nm achieves the best performance in terms of °Brix error. CNN models attain the °Brix error of ± 0.552, smaller than ± 0.597 using Feedforward Neural Network (FNN). Significantly, the CNN’s test results show that the minor error in the interval 0 to 10°Brix and 10 to 11°Brix are ± 0.551 and ± 0.408, these results indicate that the model would have the capability to predict if sugariness is below 10°Brix or not, which would be similar to the human tongue. These results are much better than ± 1.441 and ± 1.379 by using PCR and PLSR, respectively. Moreover, this study provides the test error in each °Brix interval within one Brix, and the results show that the test error is varied considerably within different °Brix intervals, especially on PCR and PLSR. On the other hand, FNN and CNN obtain robust results in terms of test error.

## Introduction

The fruit’s quality grading is one of the significant factors in processing agricultural products. A proper procedure for grading is beneficial to preservation and transportation and increasing the value of the products. The precise classification and reasonable price make customers willing to purchase the agriculture products. However, the farmers and dealers subjectively grade the quality and prices of fruits by the product’s size, appearance, color or sweetness, etc., not precisely because the criterion may not be unified. *Syzygium samarangense*, also known as wax apple, for example, the farmers usually determine the sweetness of the wax apple by the color of the peel. With more scarlet the color is, the more sugar content may be contained within the fruit. However, such judgment is not only time-consuming but also easily miscalculates the quality of the products. For more precise detection, auxiliary instruments, such as the °Brix meter or refractometer, have already been developed for detecting the sugar content. This kind of °Brix meter often utilizes light refraction through the liquid to observe and detect a small detecting error. Additionally, the °Brix obtained from the refractometer is a real number and the sugar content of an aqueous solution. Usually, one °Brix is 1 g of sucrose 100 g of solution.

Nevertheless, the drawback of the refracted meter is the procedure that needs to destroy the product itself to extract the juice for detection. This invasive method is not only time-consuming but also impractical for real applications in the agricultural field. Consequently, the non-destructive sweetness detection technique will become a crucial issue for measuring the quality of wax apple fruit.

In general, fruit’s non-destructive detecting methods are implemented by analyzing the reflectance spectra or the Hyperspectral Images (HSIs) of the surface of the fruit. Rather than ordinary RGB images, HSIs would have more features and be an ideal material to analyze. For instance, previous works presented that hyperspectral imaging is more suitable than conventional RGB imaging for evaluating mushroom quality^1^, and the advantages of analysis on HSIs are shown^2–4^. In the early researches, the analyzing methods including Multiple Linear Regression (MLR), Principal Component Regression (PCR), and Partial Least Square Regression (PLSR). Such as the soluble solids content of peaches could be determined using MLR^5^. The experiments apply PLSR on the reflectance of apples to detect the firmness^6^, the quality of gala apples could be measured by detecting the soluble solids and firmness using PCR^7^. PLSR was used to construct the models to predict glucose, sucrose, and fructose concentrations in the tubers of potatoes^8^. The concentration of anthocyanins, polyphenols, and sugar in grapes was determined using PLSR^9^. Previous work uses Independent Component Analysis (ICA) and PLSR to quantify sugar content in wax apple using spectra data with NIR bands (600–1098 nm)^10^. Moreover, some proposed researches focus on the nutrients in the wax apple, such as proving that the total anthocyanin content (TAC) and total phenolic compounds (TPC) were able to be detected by near-infrared spectroscopy^11^.

In recent years, deep learning is prevalent in developing non-destructive techniques for determining quality from various fruits. Such as the ripeness of strawberries could be analyzed with CNN and SVM models on HSIs^12^, and the maturity of citrus could be estimated with fluorescence spectroscopy data by performing the regression with a CNN model^13^. Furthermore, deep learning is also broadly applied in the actual farm field. Such as one of the researches proposed research uses R-CNN based model to detect and count passion fruit in orchards^14^, another implements CNN models on wheat yield forecasting^15^, and the other achieves high accuracy on grape bunch segmentation using deep neural networks^16^.

Those experimental results indicate the potential possibility of using the spectral convolutional neural network to detect the fruit quality. Therefore, take the fundamental requirements from the agricultural field and the spectral technique’s opportunity into consideration.

## Data preparation and evaluation process

**Figure 1:**
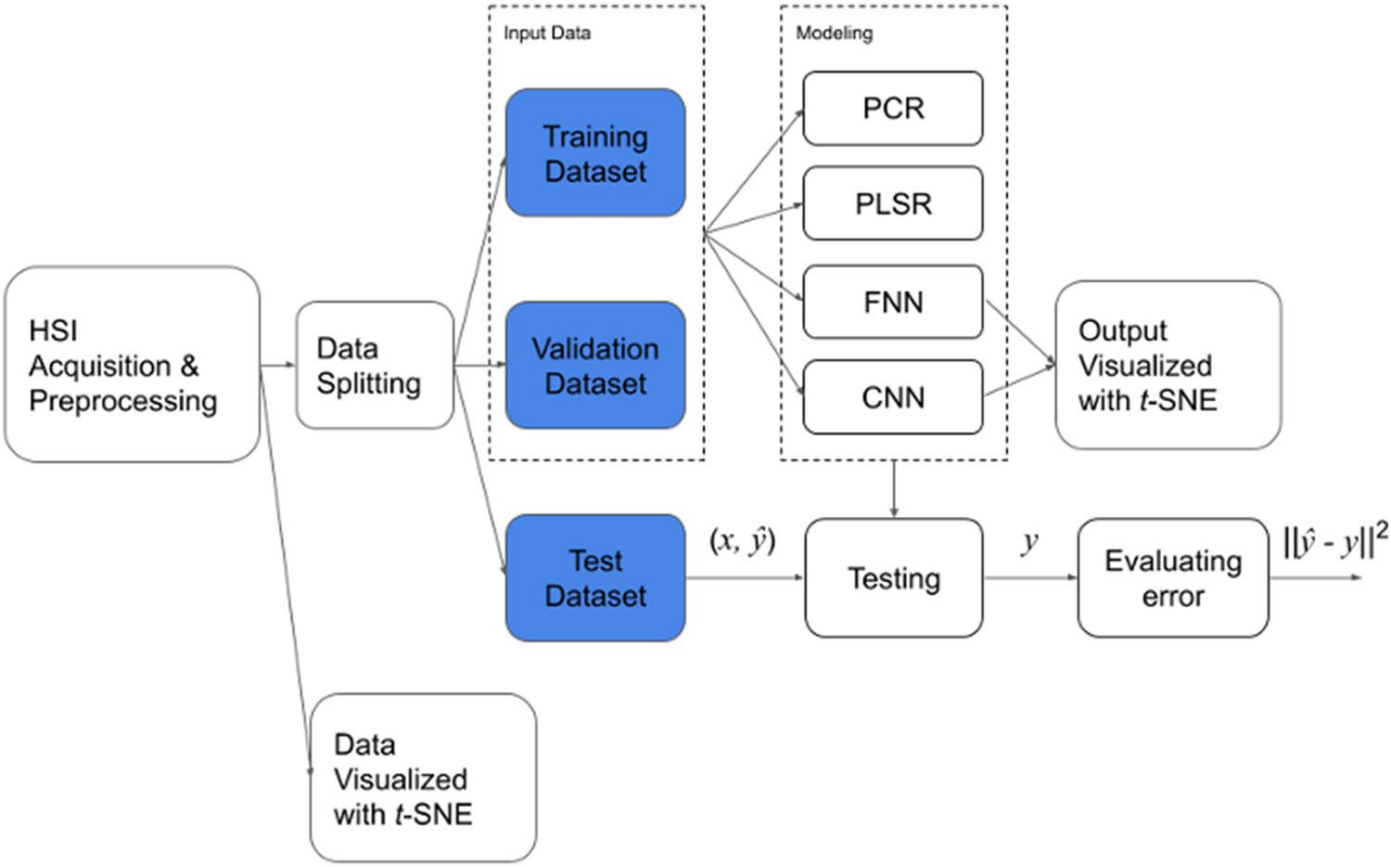
The overall workflow for evaluating the related methods in sugariness prediction.

Figure 1 shows the workflow of the entire evaluation, which consists of five steps. First, the HSIs of the bottom part of the wax apple were acquired properly and preprocessed as a 1-dimensional array for *t*-distributed Stochastic Neighbor Embedding (*t*-SNE) data visualization, FNN, PCR, and PLSR models, the 3-dimensional matrix for the 2D-CNN models. Second, *t*-SNE was used to visualize the dataset to see the distribution of data. Third, the preprocessed data was split into training, validation, and test datasets. Fourth, the FNN and CNN models were trained with training and validation datasets.

Furthermore, to review if the clustering results are better than the input samples after the deep learning process, *t*-SNE was applied on the layer’s outputs before the regression output layer. Finally, each model was evaluated with the test dataset, and the error was assessed for different models. Further information on HSIs preprocessing and modeling in this research will be introduced in the following few parts of this section.

### Samples preparation

The most important step is preparing the samples, so 136 wax apple samples (Tainung No.3, Sugar Barbie) were collected from the orchards located in Liouguei, Jiadong, Meishan, and Fengshan, Taiwan. The farmers of these wax apples are directed by experts from Fengshan Tropical Horticultural Experiment Branch (FTHEB). The wax apple samples were transported directly from different farms to a lab located at FTHEB. After wax apple samples were transferred to the lab, they were gathered and recovered to room temperature because the samples were refrigerated shipping. This step can make sure that there is no water drop on the surface of the wax apple, which may cause errors for reflectance measurement. When the samples recovered to room temperature, they were cut into small pieces. By the experience of farmers and the professional experts from FTHEB, there is more sugar content in the down part of the wax apple, and the study referred^10^ focuses on analyzing the reflectance data of the down part of the wax apple. Therefore, the pieces from the down part of the wax apple were picked up and stabbed on the blackboard to collect the HSIs data.

### HSIs data and sugariness measurement

HSIs were collected in a dark room to reduce the measurement noise with the equipment made by our team^17^. This hyperspectral system integrated two sensors for sensing visible spectroscopy (VIS) and short-wave infrared (SWIR). By the designed mechanism, the sample spectra can be collected with a spectral range from 400 to 1700 nm simultaneously. This device can obtain the complete spectral information from the surface of the wax apple, and the total size of the HSIs is $\left(W,L,\Lambda \right)$, where $W$ is width, $L$ is the length, and $\Lambda$ is the number of hyperspectral bands, which has 1367 bands from 400 to 1700 nm. Compared with spectroscopy, this device can additionally analyze the changes of spectra.

After the scanning process was done, samples were crushed to extract the juice, and the sugar content was calculated with a commercial refractometer ATAGO PAL-1. The device obtains the sugar content in terms of the index “°Brix,” which denotes the total soluble solids(TSS) in the samples. For the juice of wax-apple, the soluble solids are mainly glucose, fructose, and sucrose. Therefore, the value of °Brix obtained from wax apple juice can be regarded as the sugar content or sugariness of the sample. The average sugar content was 11.49°Brix, and the standard deviation was 1.945°Brix. These recorded values will be the labeled targets for training deep neural networks.

The preprocessing of HSIs was crucial for model training. Therefore, the HSIs were calibrated with White/Dark light calibration to obtain the reflectance $R\left(\Lambda \right)$

1 $$R\left(\Lambda \right)=\frac{{I}_{S}-{I}_{D}}{{I}_{W}-{I}_{D}}$$

where ${I}_{S}$ was the responses of the spectrometer by sensing the target, ${I}_{D}$ was the black noise of the sensor by sensing the black baffle, and ${I}_{W}$ was the responses of the spectrometer by sensing the white diffuse reflectance target.

**Figure 2:**
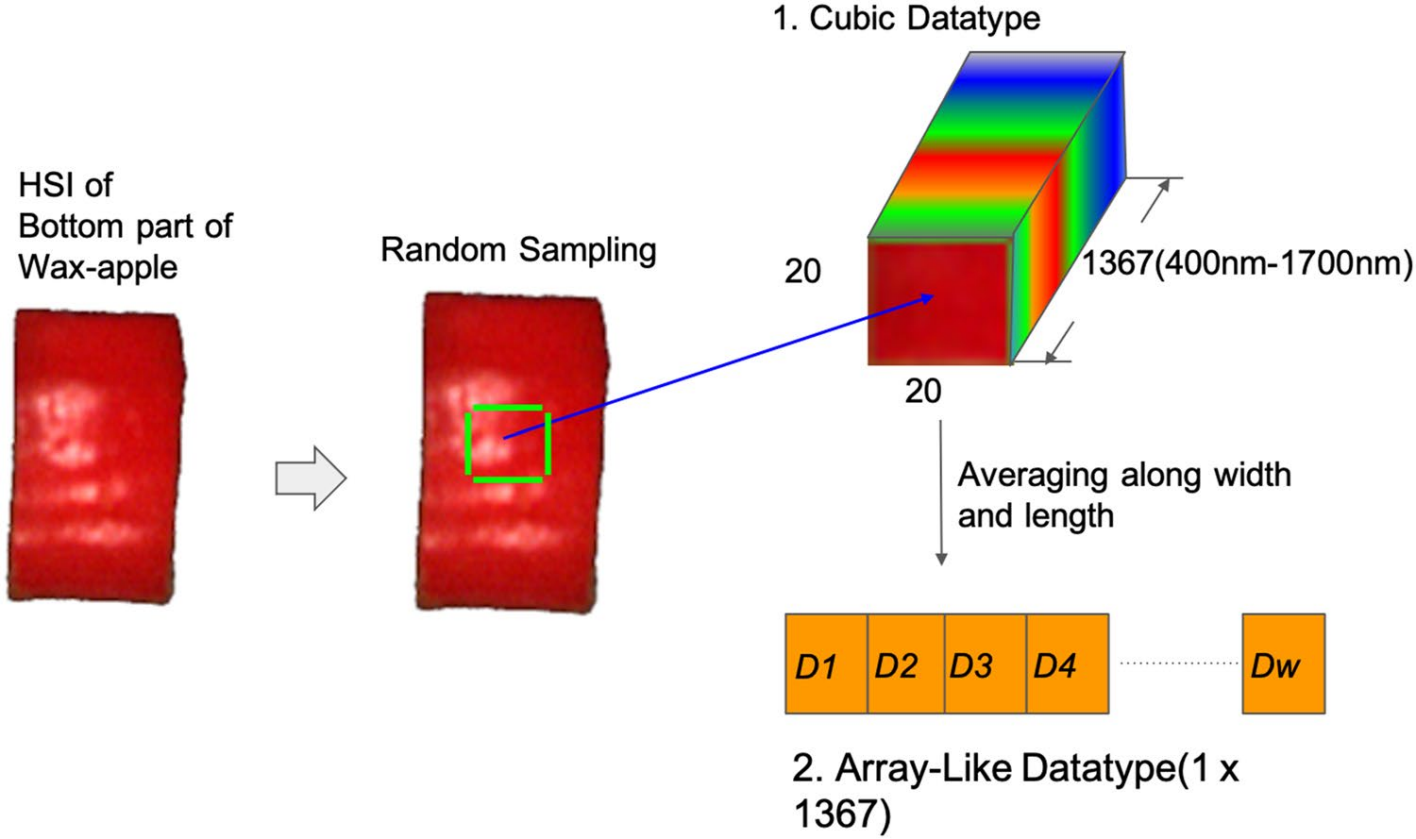
The workflow shows that the data augmentation for generating cubic hyperspectral images and the array-like spectrum averages. The smaller HSIs will be randomly sampled, and the number of the smaller HISs depends on the size of the wax apple sample. Because this study aims to analyze the spectral features of HSIs, the spatial features would not be considered in the data augmentation process.

To increase the precision of the data without distorting the signal tendency, the waveform of reflectance $R\left(\Lambda \right)$ was smooth with a Savitzky–Golay filter^18^. For data augmentation, a procedure was designed to randomly sample the HSIs of pieces of wax apple into the smaller cube with size $20\times 20\times \Lambda$. The number of smaller cubes each piece can take was according to its original size (width around 30 to 90 pixels and length around 90–150 pixels), 1034 pieces in total. The 3-D HSIs datasets will be used to train CNN models, and for *t*-SNE data visualizing and training on FNN models, PCR and PLSR, the cubic datasets were averaged to the arrays by their width and length. The entire data augmentation workflow is shown in Fig. 2. Note that in this study, the analytical strategy was focused on spectral analysis. Therefore, the proposed data augmentation method nullified the influence of spatial features of the data.

After data augmentation, the number of samples within one °Brix is shown in Table 1. First, the farmers and experts of FTHEB indicated that wax apples are qualified to merchandise if the sugar content is above 10°Brix. Therefore, it would be practical that the measurement was able to identify if the sugar content of wax apples above 10°Brix. In order to achieve that, it is essential to know the number of samples which have °Brix under 10. Second, the refractometers used for measuring sugariness had the error ± 0.5°Brix. Therefore, datasets were divided by each one °Brix. Thrid, rather than just providing an averaged error overall test data, this study would show the averaged error in each group, which would be more detailed than just an averaged error for all data. As shown in Table 1, the number of samples in °Brix 10 to 11 was less than others. Datasets were re-sampled into 218 samples in groups including "°Brix under 10", "°Brix 10 to 11", and "°Brix above 13" by random sampling to make datasets balanced. After re-sampling, the mean and the standard deviation of each group are shown in Table 2.

**Table 1:** The table shows the number of samples in each group.

Sugar content	Number of SAMPLES
°Brix ∈ [0, 10] (M:8.660, Std:0.813)	254
°Brix ∈ [10, 11] (M:10.448, Std:0.251)	162
°Brix ∈ [11, 12] (M:11.497, Std:0.232)	283
°Brix ∈ [12, 13] (M:12.526, Std:0.272)	218
°Brix ∈ [13, 17] (M:14.027, Std:0.864)	237

The M denotes the mean °Brix, and Std denotes the standard deviation of samples in each group.

**Table 2:** The table shows all the information of datasets, each 3-D and 1-D datasets consist of four different wavelength range spectra datasets where each spectra dataset is composed of five groups of random samples for °Brix under 10, °Brix 10 to 11, °Brix 11 to 12, °Brix 12 to 13 and °Brix above 13.

	Range	Size	Groups	Number of samples		
				Training	Validation	Test
3-D HSIs dataset	400–1700 nm	20 × 20 × 1367	°Brix ∈ [0, 10] (M:8.654, Std:0.823)	131	44	43
			°Brix ∈ [10, 11] (M:10.448, Std:0.251)	97	33	32
			°Brix ∈ [11, 12] (M:11.487, Std:0.231)	131	44	43
			°Brix ∈ [12, 13] (M:12.526, Std:0.272)	131	44	43
			°Brix ∈ [13, 17] (M:14.016, Std:0.888)	131	44	43
	400–1000 nm	$20\times 20\times 1053$	°Brix ∈ [0, 10] (M:8.654, Std:0.823)	131	44	43
			°Brix ∈ [10, 11] (M:10.448, Std:0.251)	97	33	32
			°Brix ∈ [11, 12] (M:11.487, Std:0.231)	131	44	43
			°Brix ∈ [12, 13] (M:12.526, Std:0.272)	131	44	43
			°Brix ∈ [13, 17] (M:14.016, Std:0.888)	131	44	43
	400–700 nm	$20\times 20\times 575$	°Brix ∈ [0, 10] (M:8.654, Std:0.823)	131	44	43
			°Brix ∈ [10, 11] (M:10.448, Std:0.251)	97	33	32
			°Brix ∈ [11, 12] (M:11.487, Std:0.231)	131	44	43
			°Brix ∈ [12, 13] (M:12.526, Std:0.272)	131	44	43
			°Brix ∈ [13, 17] (M:14.016, Std:0.888)	131	44	43
	900–1700 nm	$20\times 20\times 424$	°Brix ∈ [0, 10] (M:8.654, Std:0.823)	131	44	43
			°Brix ∈ [10, 11] (M:10.448, Std:0.251)	97	33	32
			°Brix ∈ [11, 12] (M:11.487, Std:0.231)	131	44	43
			°Brix ∈ [12, 13] (M:12.526, Std:0.272)	131	44	43
			°Brix ∈ [13, 17] (M:14.016, Std:0.888)	131	44	43
1-D spectra data	400–1700 nm	$1\times 1367$	°Brix ∈ [0, 10] (M:8.654, Std:0.823)	131	44	43
			°Brix ∈ [10, 11] (M:10.448, Std:0.251)	97	33	32
			°Brix ∈ [11, 12] (M:11.487, Std:0.231)	131	44	43
			°Brix ∈ [12, 13] (M:12.526, Std:0.272)	131	44	43
			°Brix ∈ [13, 17] (M:14.016, Std:0.888)	131	44	43
	400–1000 nm	$1\times 1053$	°Brix ∈ [0, 10] (M:8.654, Std:0.823)	131	44	43
			°Brix ∈ [10, 11] (M:10.448, Std:0.251)	97	33	32
			°Brix ∈ [11, 12] (M:11.487, Std:0.231)	131	44	43
			°Brix ∈ [12, 13] (M:12.526, Std:0.272)	131	44	43
			°Brix ∈ [13, 17] (M:14.016, Std:0.888)	131	44	43
	400–700 nm	$1\times 575$	°Brix ∈ [0, 10] (M:8.654, Std:0.823)	131	44	43
			°Brix ∈ [10, 11] (M:10.448, Std:0.251)	97	33	32
			°Brix ∈ [11, 12] (M:11.487, Std:0.231)	131	44	43
			°Brix ∈ [12, 13] (M:12.526, Std:0.272)	131	44	43
			°Brix ∈ [13, 17] (M:14.016, Std:0.888)	131	44	43
	900–1700 nm	$1\times 424$	°Brix ∈ [0, 10] (M:8.654, Std:0.823)	131	44	43
			°Brix ∈ [10, 11] (M:10.448, Std:0.251)	97	33	32
			°Brix ∈ [11, 12] (M:11.487, Std:0.231)	131	44	43
			°Brix ∈ [12, 13] (M:12.526, Std:0.272)	131	44	43
			°Brix ∈ [13, 17] (M:14.016, Std:0.888)	131	44	43

The M denotes the mean °Brix, and Std denotes the standard deviation of samples in each group.

**Figure 3:**
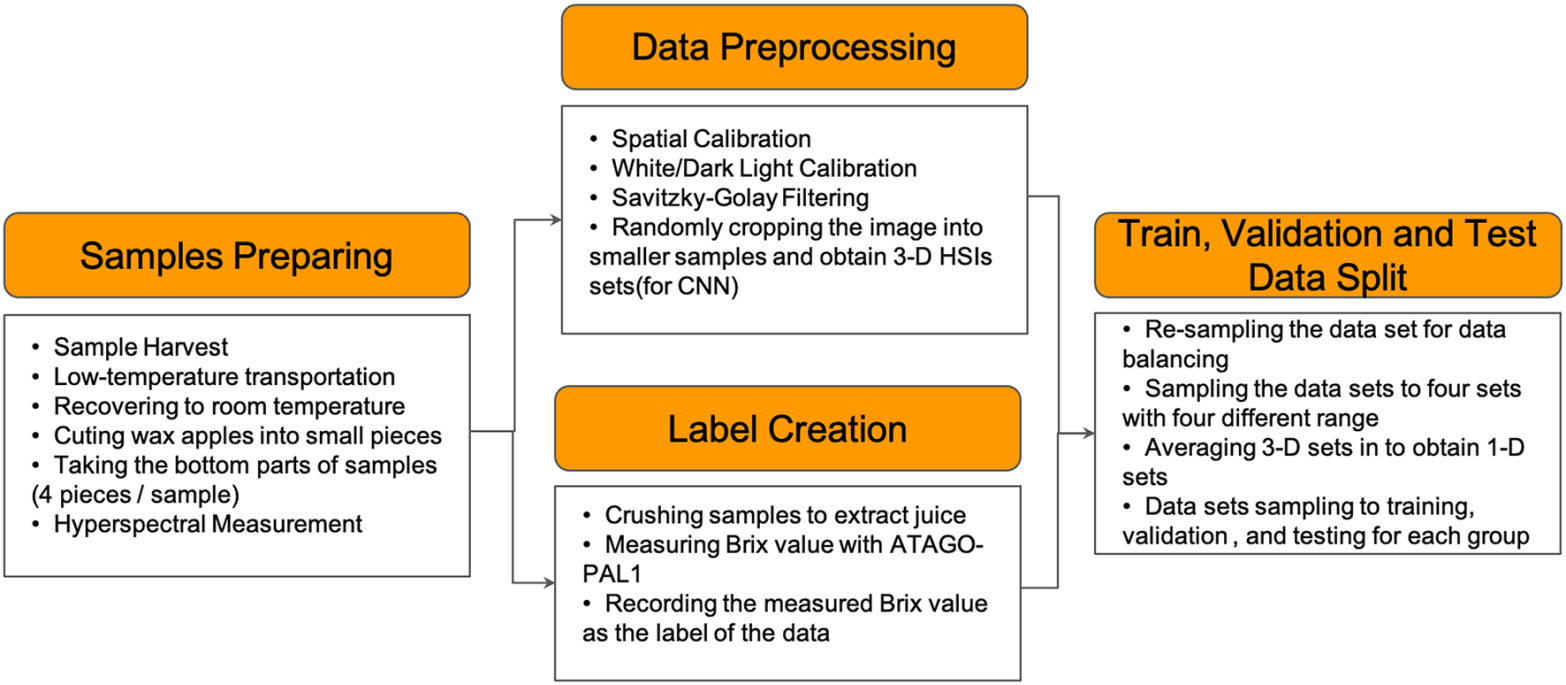
The workflow is designed for preparing experimental datasets. First, the samples are transferred to the lab under low-temperature transportation. Second, after the samples are recovered to room temperature, the samples are cut into small pieces for HSIs scanning and juice extracting. Third, the HSIs are obtained by the imaging system in a dark room. Fourth, the small samples are crushed by a small juicer to extract the juice to measure the value of sugariness, which is the data label, with a refractometer ATAGO-PAL1. Fifth, the HSIs are calibrated with spatial calibration and white/dark light calibration, then Savitzky-Golay Filtering is applied to filter the noise and smooth the waveform. Seventh, each of the HSIs cubes is randomly cropped into smaller samples for data augmentation. The detail of this step is shown in Fig. 2. Seventh, the datasets are divided into five groups, including "under °Brix 10", "°Brix 10 to 11", "°Brix 11 to12", "°Brix 12 to13", and "°Brix above 13" depending on the °Brix. The numbers of data in groups are similar for data balancing. Eighth, the 3-D HSIs datasets are sampled into four datasets with different band ranges, including 400–1700 nm (each cube with size $20\times 20\times 1367$), 400–1000 nm (each cube with size $20\times 20\times 1053$), 400–700 nm (each cube with size $20\times 20\times 575$), and 900–1700 nm (each cube with size $20\times 20\times 424$). Then, the 3-D datasets are averaged along their width and length to obtain 1-D datasets. Therefore, there had also four datasets, including 400–1700 nm (each array with size $1\times 1367$), 400–1000 nm (each array with size $1\times 1053$), 400–700 nm (each array with size $1\times 575$), and 900–1700 nm (each array with size $1\times 424$). Finally, all the datasets are sampled into "training", "validation", and "test" for modeling.

The small cube (size $20\times 20\times 1367$) HSIs datasets were quadrupled into four datasets with different band ranges, including 400–1700 nm (each cube with size $20\times 20\times 1367$), 400–1000 nm (each cube with size $20\times 20\times 1053$), 400–700 nm (each cube with size $20\times 20\times 575$), and 900–1700 nm (each cube with size $20\times 20\times 424$). The numbers 1367, 1053, 575, and 424 represent the individual bands from 400–1700 nm, 400–1000 nm, 400–700 nm, and 900–1700 nm. This study aimed to evaluate which band range has a better correlation toward making sugariness predictions. These 3-D cubes datasets will be used to train CNN models, and for *t*-SNE data visualizing and training on FNN models, PCR and PLSR, the 3-D datasets were averaged to array-like datasets by their width and length. Therefore, there had also four datasets, including 400–1700 nm (each array with size $1\times 1367$), 400–1000 nm (each array with size $1\times 1053$), 400–700 nm (each array with size $1\times 575$), and 900–1700 nm (each array with size $1\times 424$). After the datasets were prepared, they were randomly sampled into "training", "validation", and "test" for modeling. All the data and size of each set are shown in Table 2, and Fig. 3 shows the entire workflow for datasets preparation.

### Data visualization with *t*-SNE

The *t*-Distributed Stochastic Neighbor Embedding (*t*-SNE) is a non-linear machine learning algorithm proposed by van der Maaten and Hinton^19^. It is a popular manifold learning algorithm that aims to preserve the feature information when high dimensional data are visualized as low dimensional data. In t-SNE, the goal is to make the conditional probability [Eq. (2)] of high (${p}_{ij}$) and low (${q}_{ij}$) dimensional Euclidean distances as close as possible by minimizing the cost function [Eq. (3)], which is the sum of Kullback–Leibler divergences over all data points.

2 $${p}_{ij} = \frac{exp(-{||{x}_{i} - {x}_{j}||}^{2} /{ 2{\sigma }_{i}}^{2})}{{\sum }_{k\ne l}{exp({-||{x}_{k} -{ x}_{l}||}^{2} ) / {2{\sigma }_{i}}^{2}}^{2}}, { q}_{ij}=\frac{{\left(1 + {\left|\left|{y}_{i} - {y}_{j}\right|\right|}^{2}\right)}^{-1}}{{\sum }_{k\ne l}{\left(1 +{ \left|\left|{y}_{k} - {y}_{l }\right|\right|}^{2}\right)}^{-1}}$$

3 $$C={\sum }_{i}KL({P}_{i}||{Q}_{i})={\sum }_{i}{p}_{j|i}log\frac{{p}_{j|i}}{{q}_{j|i}}$$

And because σi is varied for every data point, how σi is selected is here in accordance with a fixed perplexity

4 $$Perp\left({P}_{i}\right)={2}^{H\left({P}_{i}\right)}$$

where $H\left({P}_{i}\right)$ is the Shannon Entropy ${P}_{i}$ measured in bits

5 $$H\left({P}_{i}\right)=-{\sum }_{i}{(p}_{j|i}{log}_{2}{p}_{j|i})$$

In this study, data visualization could help to do preliminary analysis. Therefore *t*-SNE was used to scale the high dimensional data $X$ (obtained from the preprocessing stage) from $d$ dimension to 2-dimensional data $Y$, where $d$ was the number of data points of whole the bands. The output $Y$ could also be considered as the basis vectors of $X$. To obtain the best output $Y$, Stochastic Gradient Descent with Momentum (SGDM) is applied to optimize the cost $C$. The equation of SGDM was defined as

6 $${Y}^{\left(t\right)}={Y}^{\left(t-1\right)}-\eta \frac{\partial C}{\partial Y}+\alpha \left(t\right)\left({Y}^{\left(t-1\right)}-{Y}^{\left(t-2\right)}\right)$$

where $t$ is the index of iteration, $\eta$ is the learning rate and $\alpha$ is the momentum.

**Figure 4:**
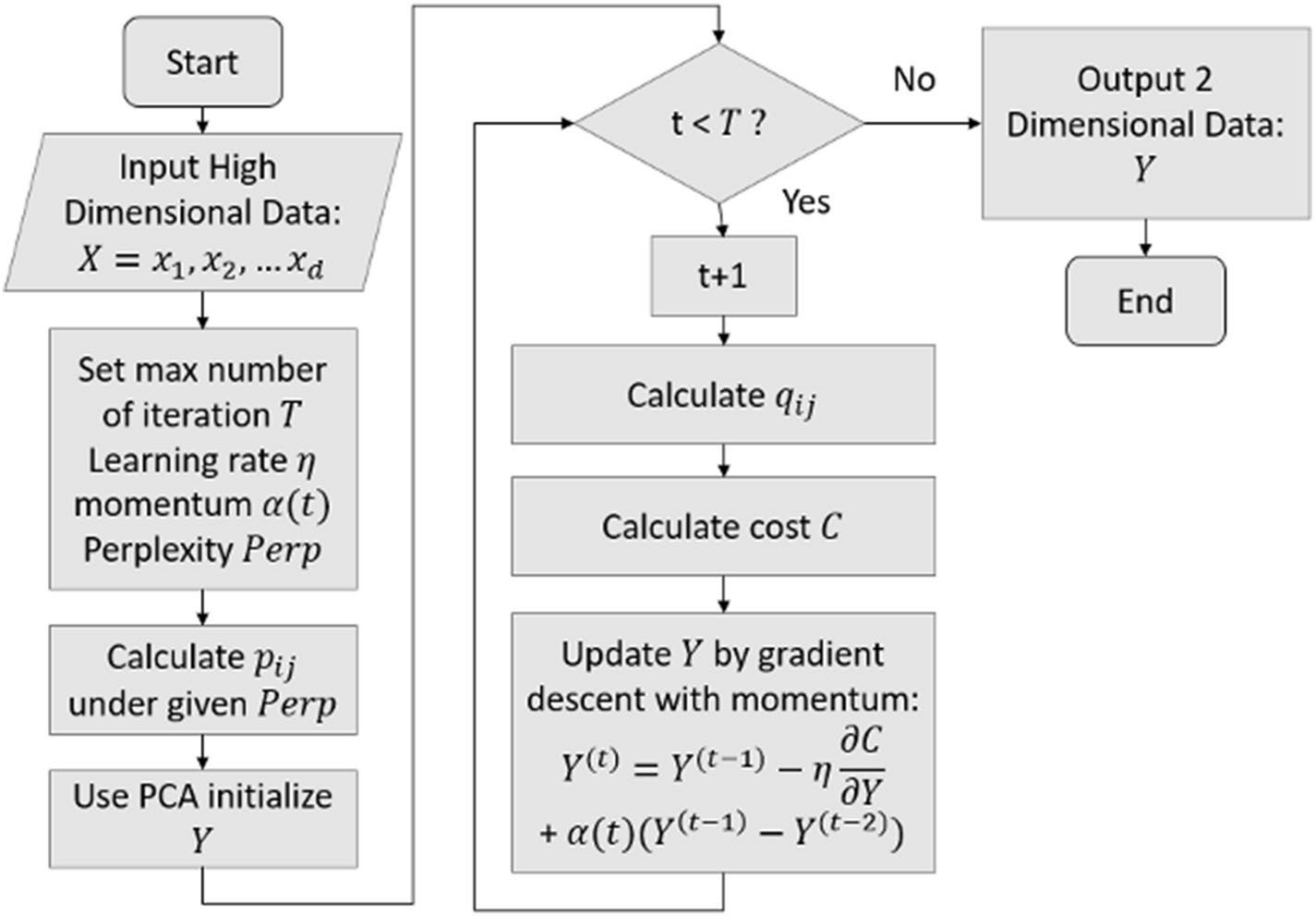
The workflow shows how this study implements *t*-SNE. The high dimensional array with spectra data ($X$) corresponding to wavelength is averaged from 3-D HSIs datasets along width and length. After setting the max number of the iteration $T$, learning rate $\eta$, momentum $\alpha \left(t\right)$, and perplexity $Perp$, the conditional probability (${p}_{ij}$) of $X$ is calculated under given $Perp$, then the Principal Component Analysis (PCA) is applied to initialize $Y$. To minimize cost $C$, the Stochastic Gradient Descent with Momentum (SGDM) is applied. When the optimization process is done, the final output $Y$ is obtained and visualized on the 2-D plane.

First, the max number of the iteration $T$, learning rate $\eta$, momentum $\alpha \left(t\right)$, and perplexity $Perp$ were set. Second, the conditional probability(${p}_{ij}$) of $X$ was calculated using Eq. (2) under a given perplexity value $Perp$, then the Principal Component Analysis (PCA) is applied to initialize $Y.$ Third, the conditional probability(${q}_{ij}$) of the initial $Y$ was calculated using Eq. (2), and the cost $C$ was obtained using Eq. (3). Fourth, if not excessing the max iteration $T$, the gradient was calculated and $Y$ was updated using Eq. (6). Finally, when the iteration was done, the final 2-dimensioanl output $Y$ was obtained and visualized on the 2-D plane. The entire workflow is shown in the Fig. 4.

**Figure 5:**
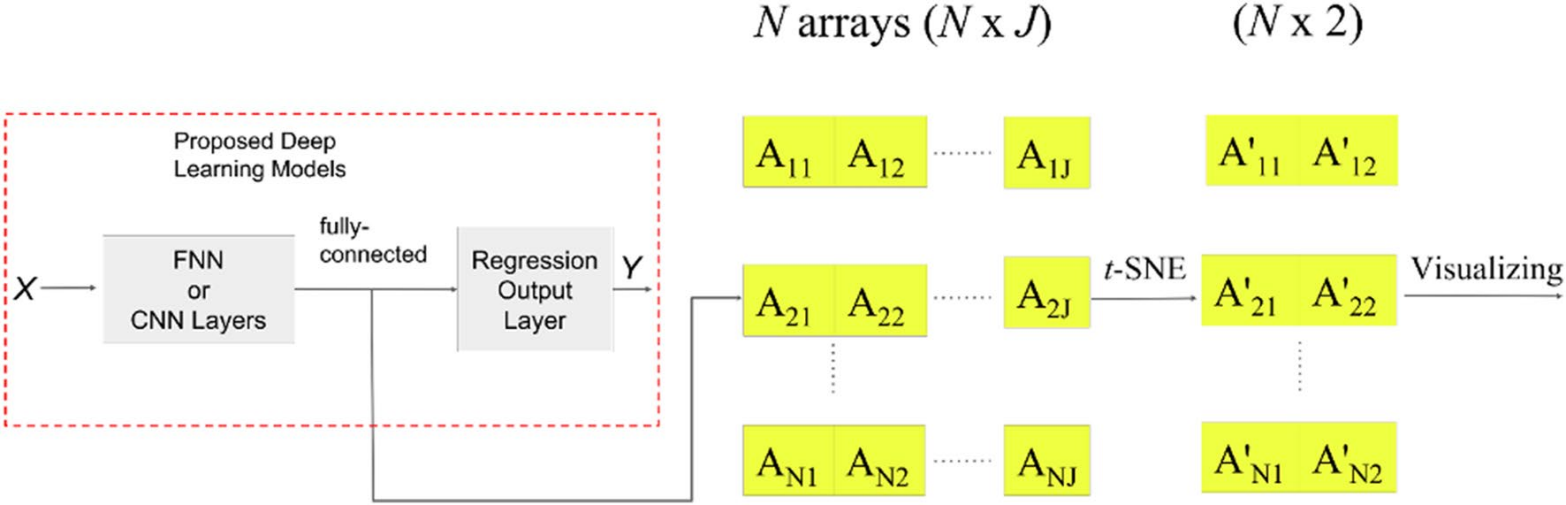
The workflow shows how *t*-SNE evaluates the learning results. First, the matrix with the size is obtained by feeding the dataset $X$ to proposed deep learning models and collecting the layer's outputs before the last regression output layer. Then the matrix is scaled to $N$ data points to visualize the *t*-SNE result. The process of *t*-SNE is as same as the process in Fig. 4.

Furthermore, to visualize the data distribution after the deep learning process to make a contrast, *t*-SNE was also used to evaluate the clustering of the outputs before the last regression output layer of proposed deep learning models. Figure 5. shows the workflow of *t*-SNE to present learning results. First of all, $X$ was the input dataset with total $N$ wax apple samples. Each data in *X* was fed into the proposed deep learning models to obtain output from the fully-connected layer to the last regression output layer. Second, the $N$ arrays with each size $1\times J$, where $J$ denoted as the number of the output of the layer before the last output layer, were composed of each output obtained by feeding each data in $X$. Finally, the matrix with size $N\times J$ was scaled to matrix $N\times 2$ using *t*-SNE as same as the process in Fig. 4, which was able to visualize in the 2-D plane.

### Hyperspectral and convolutional modeling

Two linear regression techniques and two deep learning modeling techniques were implemented on our datasets. The linear algorithms Principal Component Regression (PCR) and Partial Least Square Regression (PLSR) were applied to compare with non-linear deep learning techniques on 1-D spectrum array datasets in this research. Respectively, the proposed deep learning models were Feedforward Neural Network (FNN) and Convolutional Neural Network (CNN). These two techniques will be used on 1-D spectra datasets and 3-D HSIs datasets.

### Principal component regression and partial least square regression

PCR and PLSR were applied to our dataset to compare with deep learning methods. PCR is based on the Principal Component Analysis (PCA) and Multi-Linear Regression (MLR) approach. The main idea of the PCA is to find out the projection vector *v*^∗^ that can maximize the variation of the projected data and can be denoted as Eq. (7), where the $M$ is the covariance matrix, and *R*^*d*^ is the domain of data.

7 $${v}^{*}=\mathit{arg}\underset{v\in {R}^{d}}{\mathrm{max}}{v}^{T}Cv$$

Each vector that is found by Eq. (7) will be treated as the principal components (PCs), the first component will dominate the most significant percentage of data variance, and the following components will have the decreasing data variance for the whole signal, the PCs could be found by the singular value decomposition. All of them are linear combinations of the initial signal but not unrelated to each other. This method has the advantage of decreasing the disturbance from noise or small signals, and it is often utilized with a multidimensional reduction that retains the essential components before the following procedure. This method can eliminate the multi-collinearity because the PCs are uncorrelated. However, the PCR has the drawback that the PCs are obtained through only variables themselves. It cannot ensure that the variables correlate with the observation data, which often makes lower precision outcomes from the fitting model. Consequently, it is trendy that combining with other discriminant methods for target detecting or classifying.

PLSR^20^ was proposed to overcome the drawback of PCR; PLSR extracts the dependent variables by confirming their predictive abilities. These orthogonal factors, which are also known as latent variables (LVs), are obtained according to the correlation with the predicting variable. The general basic model of PLSR can be denoted as Eqs. (8) and (9), where $X$ is the matrix of predictors, $Y$ is a matrix of response, $T$ and $U$ are the projection of $X$ and $Y$, $P$ and $Q$ are coefficient matrices, $E$ and $F$ are the error terms. The main idea of the PLSR is to look for the proper projection that has the maximum correlation shown in Eq. (10). Different from the PCA, which extracts the PCs with a high covariance variable, the PLSR is more feasible in the condition of multi-collinearity between the variables. Thus, the number of LVs is usually less than PCs in PCR regression.

8 $$X=T{P}^{T}+E$$

9 $$Y=U{Q}^{T}+F$$

10 $$t,u=\mathrm{arg}\underset{T,U}{\mathrm{max}}Cov\left(T, U\right)$$

### Feedforward neural network

**Figure 6:**
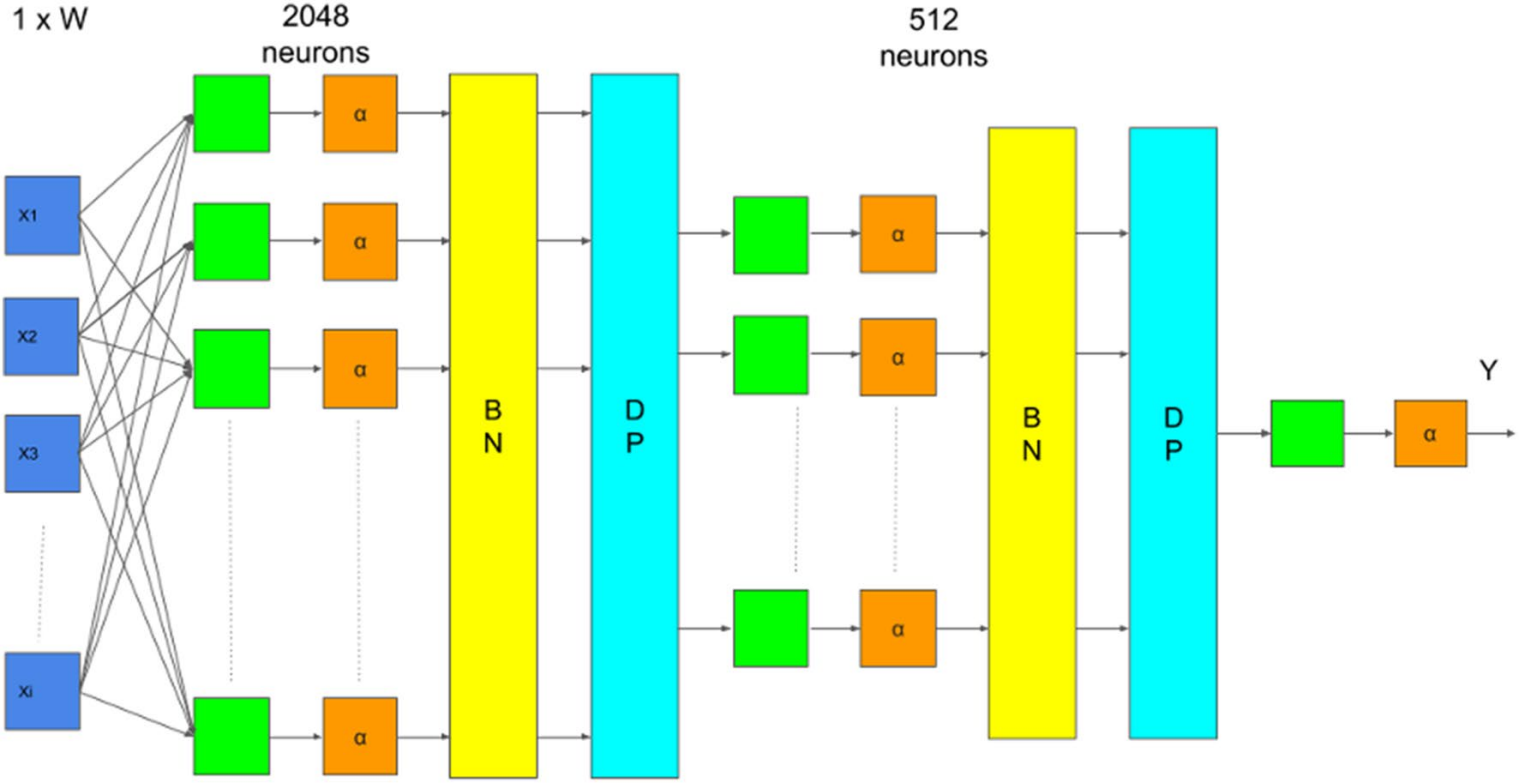
The figure shows the architecture of the proposed feedforward neural network for sugariness. Input is a 1-D spectra data with size for the number of bands. Hidden layers contain one layer with 2048 neurons and another layer with 512 neurons, each layer connected with a ReLU activation function (α), batch normalization layer (BN), and dropout layer (DP). The final output Y is the sugariness prediction result of the model.

The concept of the feedforward neural network (FNN) model was introduced by Rosenblatt in 1958^21^. In this work, FNN was applied to analyze $N\times W$ data where $N$ denotes the number of samples and $W$ denotes the number of bands. The proposed FNN model, illustrated in Fig. 6, was designed with the input layer consisting of individual hyperspectral bands of input data, hidden layers containing 2048 and 512 neurons with the batch normalization layer and dropout layer to prevent overfitting in model training, and one neuron for the output layer, each layer activated with the Rectified Linear Unit, ReLU($\alpha$), activation function. In our study, there were still some outliers in our dataset. Therefore, the Root Mean Squared Log Error (RMSLE) was used to evaluate training errors. Referring to Root Mean Squared Error (RMSE), Root Mean Squared Logarithmic Error (RMSLE) is yielded by

11 $$RMSLE=\sqrt{\frac{1}{n}{\sum }_{i}^{n}{({\mathrm{log}(Y}_{i}+1)-\mathrm{log}({Y}_{i}^{^{\prime}}+1))}^{2}}$$

where $Y$ is the predicted value, and ${Y}^{^{\prime}}$ is the target. This measure shows the ability to nullify the effects of outliers by reducing the difference between and with the natural logarithm.

### Convolutional neural networks

**Figure 7:**
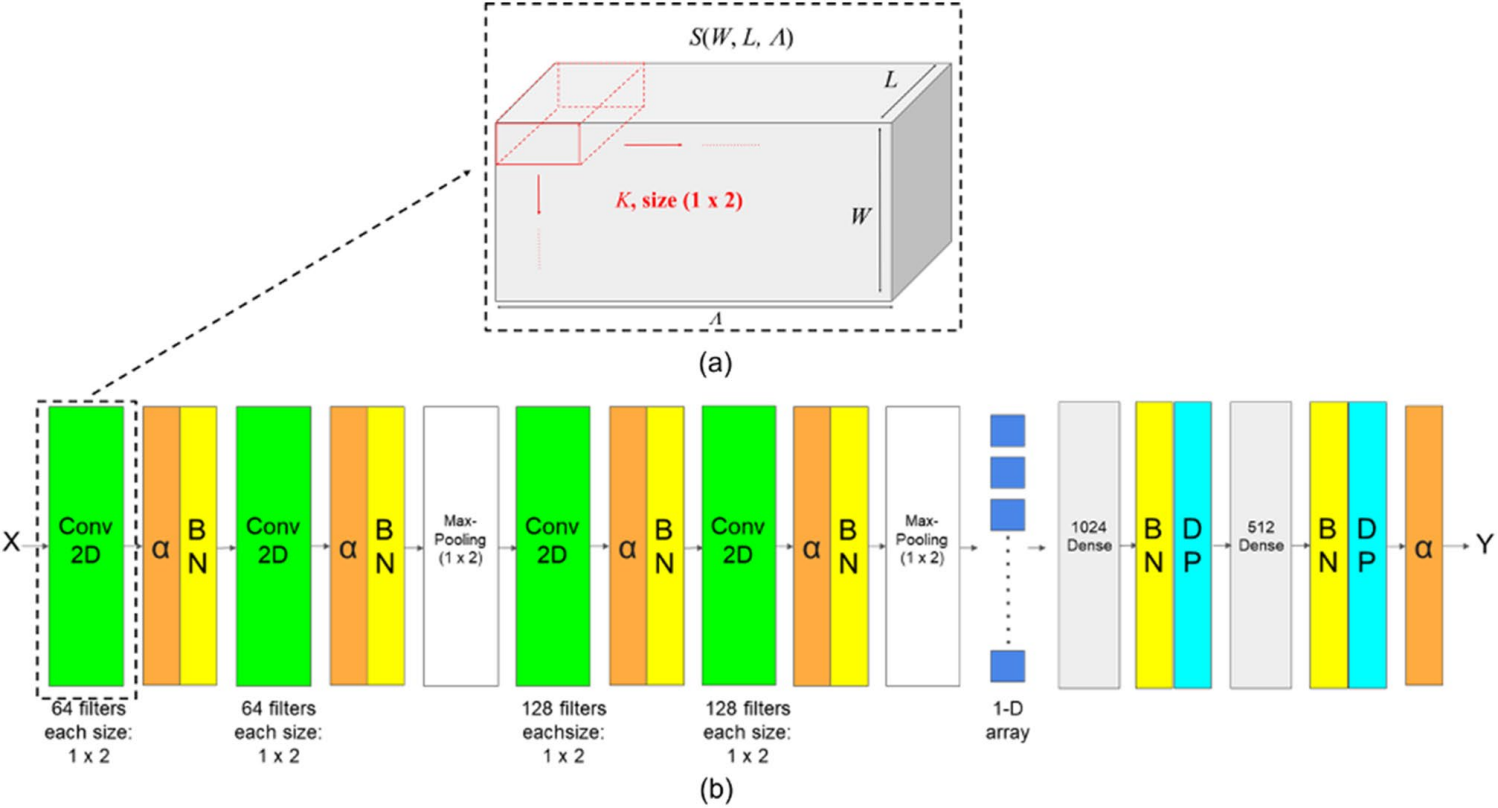
Figure (**a**) presents the proposed 2-D convolutional layer for extracting spectral features of the HSIs H with size $\left(W,L,\Lambda \right)$. Figure (**b**) shows the architecture of the proposed convolutional network for sugariness prediction. Input X is a 3-D HSIs cube sample, and the network contains four convolutional layers with a ReLU activation function. Batch normalization (BN) layers and Dropout (DP) layers are used to prevent overfitting. In the last Maxpooling layer, its output is a 1-D array obtained by calculating the maximum from each $1\times 2$ patch of the feature map then flattening. The 1-D array was then fed to fully connected layers with two dense layers with BN and DP following to regress the sugariness prediction result Y.

Convolutional Neural Network (CNN) was also one of the approaches applied to our dataset. LeCun proposed the modern CNN architecture in 1998^22^. Its strong ability to extract spatial features makes it achieve state-of-the-art performance in computer vision tasks. However, in this study, instead of extracting spatial features, the 2-D CNN model was used to extract spectral features from 3-D HSIs. The proposed 2-D convolutional layer to extract spectral features of HSIs with one kernel is shown in Fig. 7a. To extract spectral features, the cube with size $\left(W,L,\Lambda \right)$ would be rotated in the convolution process, where $W$ denotes the width of HSIs with constant value 20, $L$ denotes the length of HSIs with constant value 20, and $\Lambda$ denotes the number of bands of a hyperspectral image. The 2-D convolutional layer was designed to conduct the convolution along the surface $\left(W,\Lambda \right)$ and consider the length of HSIs *L* as the channels and apply the kernel to all the channels. Therefore, a 2-D feature map represented as spectral features would be obtained after the convolution of HSIs and a single kernel. The value of the 2-D feature map at the position $\left(\omega ,l,\lambda \right)$ denoted as G $\left(\omega ,l,\lambda \right)$ is given by

12 $$G\left(\omega ,l,\lambda \right)=\sum_{{\omega }^{^{\prime}}=0}^{{k}_{\omega }-1}\sum_{{\lambda }^{^{\prime}}=0}^{{k}_{\lambda }-1}K\left({\omega }^{^{\prime}},{\lambda }^{^{\prime}}\right)S\left(\omega -{\omega }^{^{\prime}},l,\lambda -{\lambda }^{^{\prime}}\right)$$

where $\omega^{\prime}$ and ${\lambda }^{^{\prime}}$ are the index of the kernel, ${k}_{\omega }$ and ${k}_{\lambda }$ are the width and length of the kernel, $\omega$, $l$, and $\lambda$ are the position of HSIs, $S$ is the HSIs, and $K$ is a kernel in the proposed 2-D convolutional layer. Optimizing convolutional network was used to find the optimal kernel parameters which provide high impulse response to local spectral features. The calculated response was feasible to apply Maxpooling to preserve the spectral features with high response. By applying the backpropagation algorithm, the kernel parameters were updated to reduce the regression error for the downstream task. Based on this solution, the spectral features which map to the features of sugariness could be found.

The entire architecture of the model is shown in Fig. 7b. A model was designed to extract spectral features from the HSIs, which had four convolutional layers with filter size $1\times 2$(${k}_{\omega }$ = 1, and ${k}_{\lambda }$ = 2) and two Maxpooling layers with size $1\times 2$ and fully connected layers with $1\times 1$ dense layer for output, all the convolutional layers with Batch Normalization (BN) and Rectified Linear Unit (ReLU) as the non-linear activation function. As mentioned earlier, the convolutional layer was designed to extract spectral features, and the Maxpooling layers were also designed to extract spectral features. Assume after conducting the convolution, normalization, and activation, the cubic feature map with size $\left({W}^{^{\prime}},{L}^{^{\prime}},{\Lambda }^{\mathrm{^{\prime}}}\right)$ was obtained. Then the Maxpooling layers would select the maximum from each $1\times 2$ patch on the space $\left({W}^{^{\prime}},{\Lambda }^{\mathrm{^{\prime}}}\right)$ in order to preserve the spectral features. Therefore, the size of the feature map after pooling would be $\left(\frac{{W}^{^{\prime}}}{2},{L}^{^{\prime}},\frac{{\Lambda }^{^{\prime}}}{2}\right)$. After extracting features, the output feature map obtained from the last Maxpooling layer is flattened to a 1-D array and fed into fully connected layers. Finally, the final activation layer was also a ReLU function. It gave a °Brix value of the wax apple sample, and the error is evaluated with Root Mean Squared Logarithmic Error (RMSLE) in Eq. (10).

### Ethical approval

The collection of the cultivated plants of this study complied with the relevant institutional, national, and international guidelines and legislation. The farmers permitted the transaction of plants in studying usage. If request, the datasets of HSIs of wax apples will be available.

## Results

### Evaluation by data visualization

**Figure 8:**
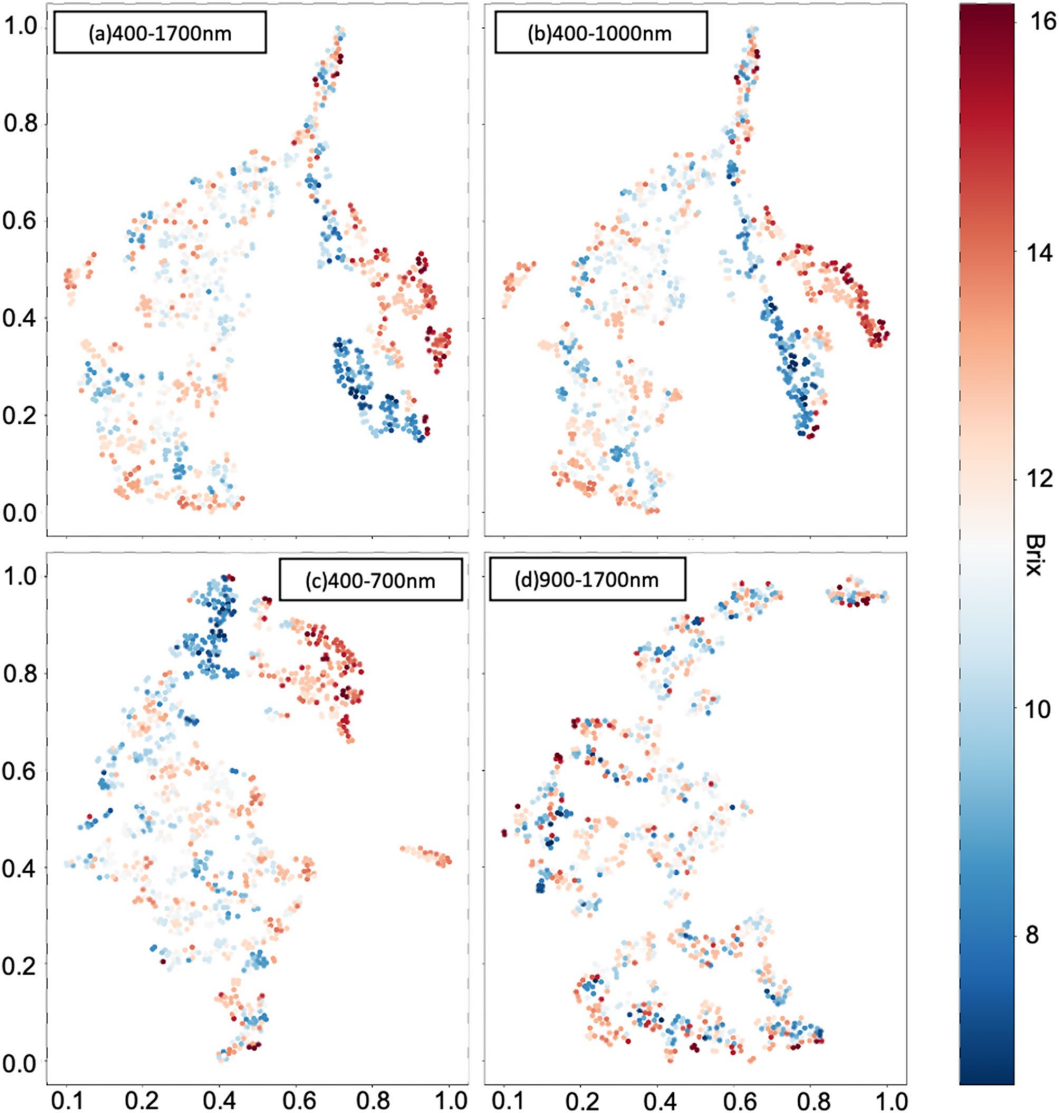
The figure shows the results of *t*-SNE on the four datasets, (**a**–**d**) are the datasets of wavelength range 400–1700 nm, 400–1000 nm, 400–700 nm, and 900–1700 nm, the color bar in the right side denoted the °Brix value of each data point. All the *t*-SNE results are obtained with the perplexity value 20, the 30,000 iteration times, and the learning rate 1000. The horizontal and vertical coordinates are the coordinate of data points obtained from dimension reduction using *t*-SNE.

In the experiments, *t*-SNE was applied to reduce the dimensionality of HSIs and visualize the distribution of the datasets, the workflow of *t*-SNE shown in Fig. 4. The dataset $X$ used to make dimension reduction were the array datasets averaged from 3-D HSIs, because it would be time-consuming, and the results were not too much different with 3-D HSIs. The entire dataset contained 932 reflectance spectral data arrays for different bands. Before starting the *t*-SNE algorithm in the experiment, the Principal Component Analysis (PCA) was used to initialize the value for dimension reduction results $Y$. Then the *t*-SNE models were trained with the perplexity value 20, the 30,000 iteration times, and the learning rate 1000 with SGDM. Figure 8 shows the visualization results of four different datasets.

Figure 8a–c show the *t*-SNE results of datasets with wavelength 400–1700 nm, 400–1000 nm, and 900–1700 nm, although the data points of sugariness above 13°Brix and under 10°Brix seem to be clustered, the clusters of data points with sugar content between 13°Brix and 10°Brix seem to be ambiguous. On the other hand, the clusters of *t*-SNE results shown in Fig. 8d are much more ambiguous than the different datasets. Therefore, it would indicate that the spectra data of wavelength 400–1000 nm were more significant than the spectra data of wavelength 900–1700 nm to regress the sugariness value. This *t*-SNE result reveals that the spectra data of visible spectroscopy (VIS) bands could contain more significant features toward predicting sugariness than the near-infrared (NIR) spectroscopy bands.

### Evaluation over different models

The weights and kernels in FNN and CNN models were initialized with Glorot initialization^23^ and regularized with L2 regularization^24^ with regularization parameter $3\times {10}^{-5}$ while training. Adam optimizer^25^ was used with mini-batch 128 and 3000 epochs for all the models to optimize the weights of each layer of FNN models and kernels of CNN models. Learning rate decay method applied in the experiments for optimizing models, the initial learning rate value for first epoch was $1\times {10}^{-3}$, and an exponential decay ${lr}_{t}={lr}_{t-1}*{e}^{\left(-k*t\right)}$ ($lr$ denotes as the learning rate, $t$ denotes as the epoch number, $k$ denoted as a constant of $1\times {10}^{-4}$) was applied for following epochs.

**Figure 9:**
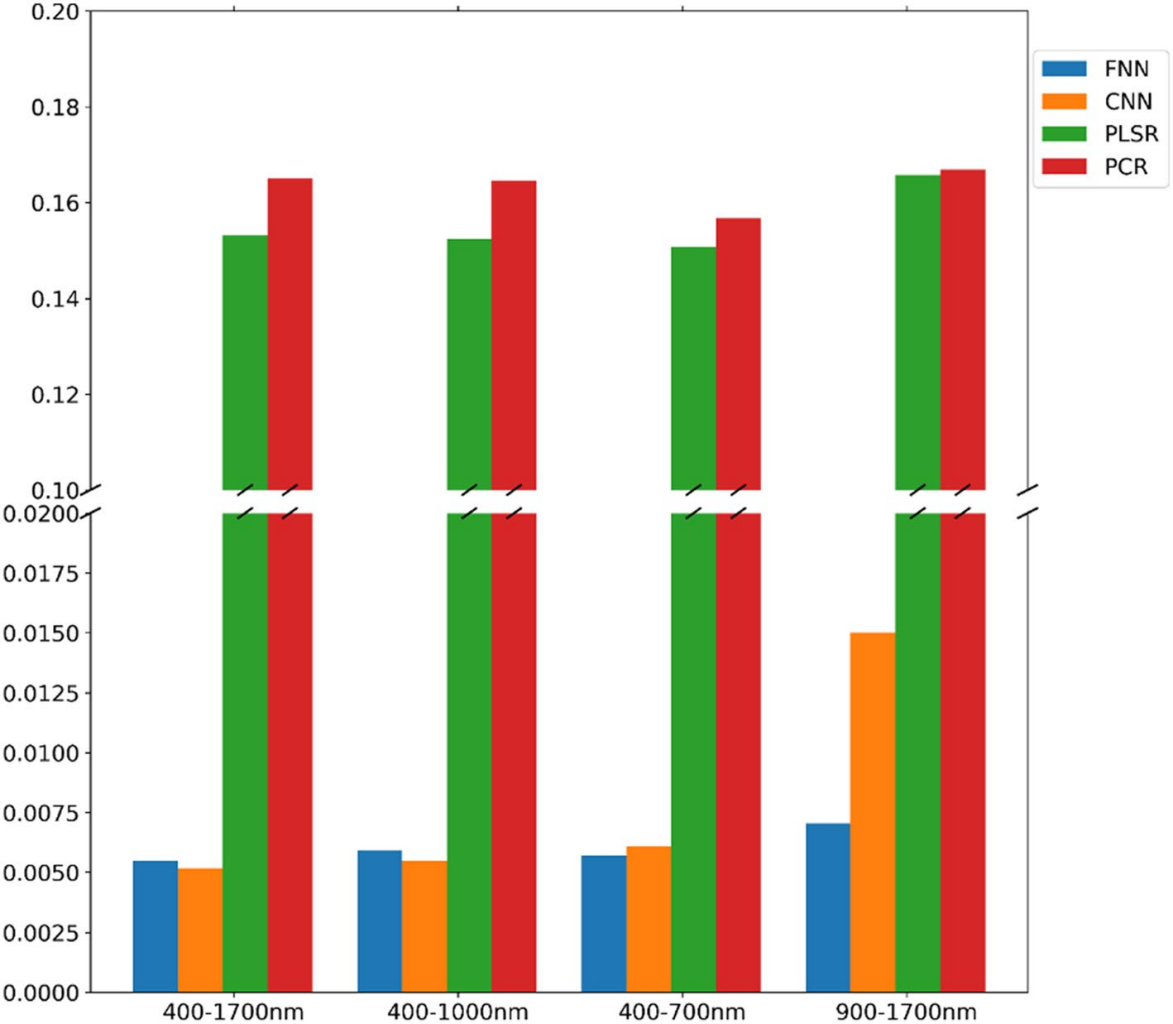
The bar chart shows the validation loss (RMSLE) of each proposed model. In the optimization process, the weights with the smallest validation loss are recorded as the best sets. This validation error presented in the chart indicates that FNN and CNN would have superior performance compared with PCR and PLSR on the datasets.

The bar chart Fig. 9 shows the RMSLE of each method on the validation set. The trained weights were chosen at the step with the smallest validation loss in the optimization process for testing each of the deep learning models. The minimum of the validation loss of each FNN model for 400–1700 nm, 400–1000 nm, 400–700 nm, and 900–1700 nm were 0.005463 at step 2734, 0.005917 at step 2736, 0.005707 at step 2540, and 0.007042 at step 2980. The model for 400–1700 nm had the lowest loss, and the model for 900–1700 nm had the highest loss, so it was expectable that the FNN model for 400–1700 nm would have better performance on the test dataset than the other FNN models. There were also four models for CNN. The minimum of validation loss (RMSLE) of the 400–1700 nm model was 0.005165 at step 2844, 0.005478 at step 2732 for the model of 400–1000 nm, 0.006077 at step 2092 for the model of 400-700 nm, and 0.015015 at the step 2994 for the model of 900–1700 nm. As same as the results of FN models, the CNN model for 400–1700 nm had the lowest loss, and the model for 900–1700 nm had the highest loss. Comparing the results of FN with the results of CNN models, the CNN models would have better performance than FNN models. The deep learning modeling was implemented using the Keras^26^ and Tensorflow backend^27^ on an NVIDIA RTX 3090 GPU.

PCR and PLSR were used to make a comparison with deep learning methods on the same training datasets. The PCR result showed that the validation loss (RMSLE) on four validation datasets was 0.165022 for the 400–1700 nm model, 0.164529 for the 400–1000 nm model, 0.156733 for the 400–700 nm model, and 0.166839 for the 900–1700 nm model. On the other hand, validation losses of PLSR were 0.153204, 0.152438, 0.150596, and 0.16576 on four different datasets. These results indicate that the PLSR method would perform better than PCR on the collected datasets, and deep learning methods would be much better than PCR and PLSR.

**Figure 10:**
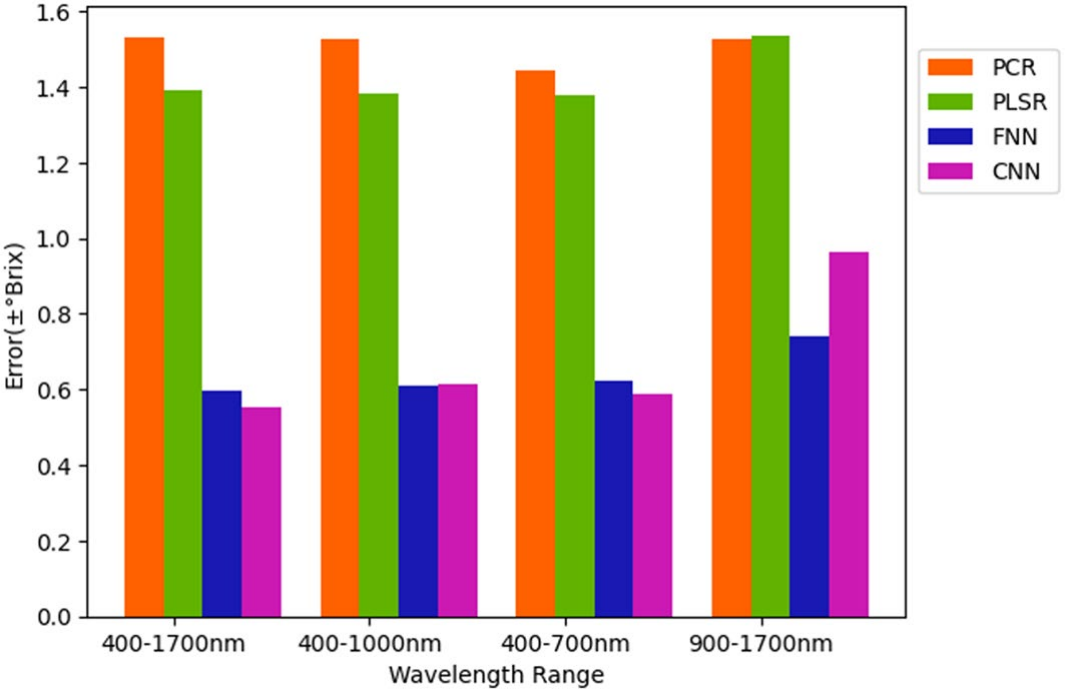
The bar chart shows that the test result of each model. The test results of FNN and CNN have more minor errors than PCR and PLSR. It indicates that the performance of non-linear deep learning models is better than linear models on the datasets.

The test results of °Brix of PCR, PLSR, FNN, and CNN models in each interval are shown in Table 3. First of all, although PCR and PLSR showed their good capability in predicting sugar content in °Brix 11 to 12, the test errors in other °Brix intervals and averaged test errors were not competitive with errors of FNN and CNN models. This phenomenon indicated that the PCR and PLSR could have an overfitting problem on this dataset and improperly detect sugar content of wax apple. The FNN models had the average error with ± 0.597°Brix on the dataset 400–1700 nm, 400–1000 nm with ± 0.610°Brix, 400–700 nm with ± 0.623°Brix, and 900–1700 nm with ± 0.739°Brix. The test results showed that FNN models trained using the dataset of wavelength range 400–1700 nm had adequate performance, and wavelength range 900–1700 nm would not correlate with sugar content prediction using FNN. The test results of CNN models were also similar to the test results of FNN models. The test error was ± 0.552°Brix on the dataset of wavelength 400–1700 nm, ± 0.616°Brix on the dataset of wavelength 400–1000 nm, ± 0.587°Brix on the dataset of wavelength 400–700 nm, and ± 0.7396°Brix on a dataset of wavelength 900–1700 nm. The test results of CNN models were superior to FNN models. Both FNN and CNN results showed that spectra data corresponding to 400–1700 nm, 400–1000 nm, and 400–700 had better correlation than data corresponding to 900–1700 nm. This conclusion indicated that VIS bands with wavelength 400–700 nm might be more correlative to predict sugar content than NIR bands with wavelength 900–1700 nm. However, the combination of VIS bands and NIR bands would help the FNN and CNN models get a better sugariness prediction result rather than only with VIS bands or NIR bands. On the other hand, the minimal error of PCR and PLSR were ± 1.444°Brix and ± 1.379°Brix on the dataset of wavelength 400–700 nm, but both of them had their best (± 0.180°Brix and ± 0.218°Brix) in °Brix interval 11–12 on 900–1700 nm. Therefore, PCR and PLSR were more severe with overfitting problems on predicting sugar content using spectra data with wavelength range 900–1700 nm than the other band range. The averaged test error of each learning method on different band ranges was presented in Fig. 10. The outcomes of test data indicated that the deep learning methods FNN and CNN were more remarkable than PCR and PLSR on predicting sugariness. These results were also reflected in validation results.

**Table 3:** The table shows all the test errors in different band ranges and °Brix value intervals.

Method	Band range	Averaged sugar content error in each interval(± °Brix)					
		0–10	10–11	11–12	12–13	Above 13	All
		43 samples	32 samples	43 samples	43 samples	43 samples	162 samples
PCR	400–1700 nm	2.727	1.061	0.218	1.103	2.430	1.532
	400–1000 nm	2.678	1.051	0.233	1.107	2.441	1.526
	400–700 nm	2.504	0.942	0.339	1.018	2.287	1.444
	900–1700 nm	2.797	1.063	0.180	1.080	2.394	1.526
PLSR	400–1700 nm	2.449	0.927	0.360	0.979	2.119	1.391
	400–1000 nm	2.410	0.954	0.371	0.965	2.098	1.382
	400–700 nm	2.405	0.958	0.375	0.218	2.369	1.379
	900–1700 nm	2.821	1.049	0.218	1.100	2.369	1.536
FNN	**400–1700 nm**	**0.642**	**0.646**	**0.511**	**0.564**	**0.637**	**0.597**
	400–1000 nm	0.625	0.633	0.437	0.474	0.889	0.610
	400–700 nm	0.732	0.693	0.355	0.463	0.892	0.623
	900–1700 nm	0.710	0.656	0.576	0.701	1.032	0.732
CNN	**400–1700 nm**	**0.609**	**0.474**	**0.412**	**0.421**	**0.660**	**0.552**
	400–1000 nm	0.627	0.487	0.451	0.516	0.965	0.616
	**400–700 nm**	**0.551**	**0.408**	**0.462**	**0.555**	**0.914**	**0.587**
	900–1700 nm	1.519	0.725	0.496	0.480	1.543	0.965

The average test results of FNN and CNN indicate that the models have a decent complexity for datasets. On the other hand, PCR and PLSR would have underfitting problems on the datasets. The better average sugar content for all the samples is shown in bold. The marked results have the error below ± 0.6°Brix.

### Evaluation of the learning results by visualizing the outputs before the last layer

**Figure 11:**
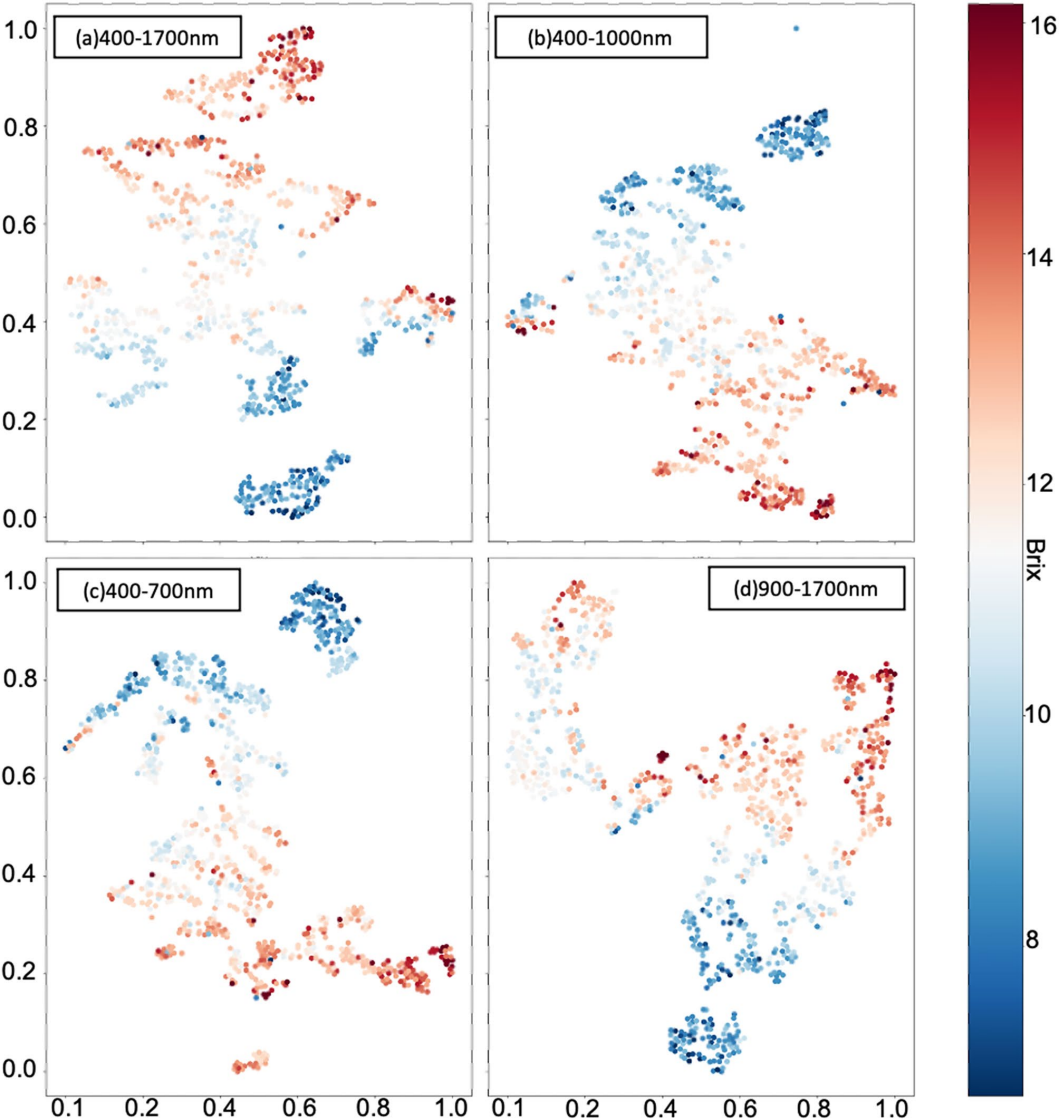
The figure shows the result of *t*-SNE on the outputs obtained by feeding the four datasets to FNN models, (**a**–**d**) are the datasets of wavelength range 400–1700 nm, 400–1000 nm, 400–700 nm, and 900–1700 nm. The color bar on the right side is denoted as the °Brix of each data point. Compared to *t*-SNE results on original datasets, figures (**a**–**c**) generally show a fine color gradation from deep red to deep blue. Relatively figure (**d**) contains more vague clusters than others. The horizontal and vertical coordinates are the coordinate of the basis vectors, which are obtained from dimension reduction using *t*-SNE.

**Figure 12:**
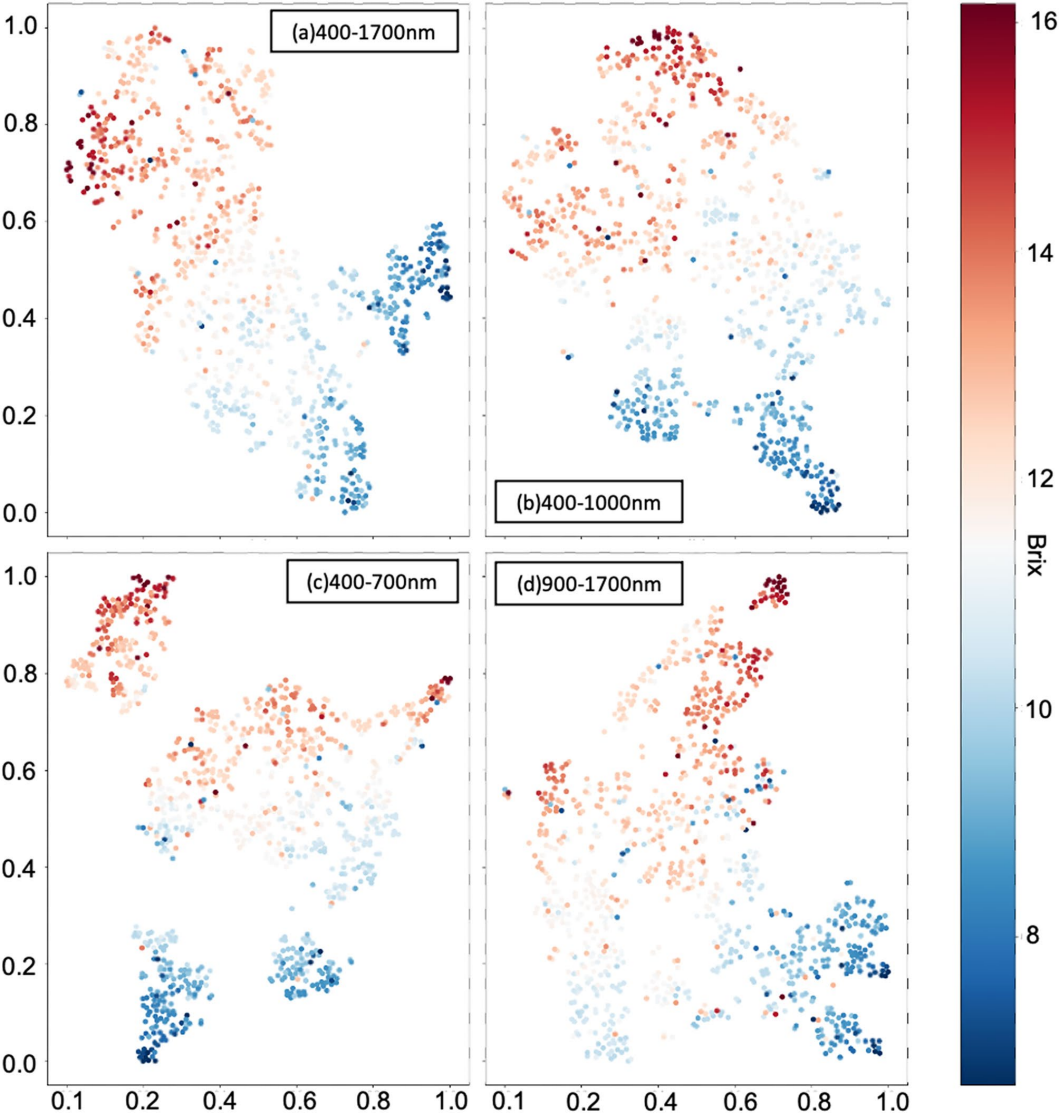
The figure shows the *t*-SNE results on the outputs obtained by feeding the four datasets to CNN models are visualized in (**a**–**d**) corresponding to wavelength range 400–1700 nm, 400–1000 nm, 400–700 nm, and 900–1700 nm. The color bar on the right side is represented as the °Brix of each data point. Compared to *t*-SNE results on original datasets, figures (**a**–**d**) generally show the better color gradation from deep red to deep blue. Although there are some outliers contained in clusters in figure (**d**), the *t*-SNE visualization results in the figure (**a**–**d**) are much better than ones in Fig. 8a–d. The horizontal and vertical coordinates are referred to as the coordinate of basis vectors obtained from dimension reduction using *t*-SNE.

Visualization was also a decent way to evaluate the proposed deep learning models. Therefore, *t*-SNE was applied to visualize the outputs of the layer before the last regression output layer. As same as the previous implementation of *t*-SNE, the PCA was used as the initial value for dimension reduction results, the perplexity value was set 20, the 30,000 iteration times, and the learning rate 1000 were used in this implementation for SGDM. The workflow is shown in Fig. 5. Figures 11 and 12 show the final visualization results of MLP and CNN models corresponding to four different wavelength ranges.

For MLP models, the output size of the layer before the last output layer was 512. Therefore, the output was an array with size $1\times 512$. Then all the outputs from feeding all the datasets ($N\times 512)$ were collected as the input data $X$ for *t*-SNE, and scaled to it’s basis vectors $Y$ which were visualized in the Fig. 11. In the Fig. 11a–c show the final evaluation results corresponding to wavelength range 400–1700 nm, 400–1000 nm, and 400–700 nm and although there are still some vague clusters, each of the results presents the proper color gradation generally from deep red to deep blue with respect to high sugar content value to low sugar content value, which represents the acceptable learning ability of MLP models on those datasets. Although the visualization results were better than the *t*-SNE results of the original data, ambiguous clusters are still contained in Fig. 11d. Therefore, this result elaborates that wavelength 900–1700 nm spectra data would not be advantageous for sugar content prediction.

On the other hand, Fig. 12a–d visualize the *t*-SNE results on the outputs of the layer before the last output layer of CNN models corresponding to the wavelength range 400–1700 nm, 400–1000 nm, 400–700 nm, and 900–1700 nm. According to the CNN architecture shown in Fig. 7b, each of the data points in Fig. 12 was also obtained by scaling output from $1\times 512$ to the array with size $1\times 2$ using *t*-SNE. The *t*-SNE visualization results gave the fine gradational effect shown in Fig. 12a–d. However, more outliers appeared in clusters of *t*-SNE results in Fig. 12d. These results showed that the CNN models could learn features toward the sugar content prediction by showing better clustering results on outputs before the last regression output layer than the ones on original data.

## Conclusions and discussion

This study aims to predict the sugariness of *Syzygium samarangense* from hyperspectral images using deep learning methods. The HSIs were collected from the bottom part of the wax apple and adequately processed to 1-D spectra datasets and 3-D HSIs datasets. The spectra data were adequately sampled and divided into 400–1700 nm, 400–1000 nm, 400–700 nm, and 900–1700 nm to facilitate learning from the corrected datasets, then *t*-SNE was used to evaluate the performance of the spectral band. In the modeling stage, the FNN and CNN models were designed for analyzing the HSIs datasets. For comparing with deep learning methods, PCR and PLSR were used on the same datasets. The best results of FNN and CNN were 0.552 and 0.597 (± °Brix) on datasets with 400–1700 nm, relatively, PCR and PLSR had their best result on datasets with 400–700 nm, which were 1.379 and 1.444 (± °Brix). In this study, FNN and CNN models outperformed PCR and PLSR models. Otherwise, all their well-performed results were obtained with the spectra data with a combination of VIS bands and NIR bands, and the results with only using NIR bands (900–1700 nm) were not that acceptable. Therefore, this study revealed that VIS bands would be more critical in predicting sugar content, but it would be better with the combination of VIS bands and NIR bands. This result showed the potential performance on acquiring sugar content of wax apple with spectra data corresponding to VIS bands and NIR bands using a deep learning model. However, only the NIR bands would not be recommended. Moreover, the improved *t*-SNE results on the final regression outputs before the last layer of proposed deep learning models show that FNN and CNN models would adequately learn how to predict sugariness from the HSIs datasets.

Compared with the previous study^10^, this paper provided the average error and the error in each °Brix interval within one °Brix. Although their works had good prediction results with total SEV = 0.381 and 0.426°Brix for ICA and PLSR using only NIR bands, this study indicated that the error would be significantly different within different sugar content intervals. Therefore, this study which provided errors of different sugar content intervals, would be more detailed than theirs.

It will be significant to select proper bands for fabricating a portable device to detect sugariness in future work. The light source with VIS bands and the CNN model would be a good start for constructing a mobile device because the light source with VIS bands is more easily available than NIR bands. Moreover, although without NIR bands, the combination of the CNN model with VIS bands shows adequate performance on prediction sugariness of wax apple in this study with the minor error in interval 0 to 10°Brix and 10 to 11°Brix are ± 0.551 and ± 0.408. These results indicate that the CNN model using VIS spectra data would have the capability to predict if sugariness is below 10°Brix or not. This would be similar to the human’s eyes because when the wax apples get ripe, the wax apples gain more sugar and become more scarlet than before. Therefore, the human’s eyes could roughly detect the sugar contents by the color of the wax apples. However, this study also indicates that if both the visible and invisible spectral features are considered, it would better predict the sugariness of wax apples.
